# The Injuries Incidental to Athletic Exercises

**Published:** 1908-09-19

**Authors:** R. H. A. Whitelocke

**Affiliations:** Hon. Surgeon to the Radcliffe Infirmary, Oxford; Lichfield Lecturer in Surgery in the University.


### Hospital Clinics.

### THE INJURIES INCIDENTAL TO ATHLETIC EXERCISES.

By R. H. A. WHITELOCKE, M.D. (Edin.), F.R.C.S., Hon. Surgeon to the Radcliffe Infirmary, Oxford; Lichfield Lecturer in Surgery in the University.

(Report of a Lecture at the Medical Graduates' College and Polyclinic.

The injuries incidental to athletic exercises are many and varied. It would seem impossible to cover the ground in a single lecture, and I shall, therefore, ask you to allow me to refer to some of those most commonly met with.

Perhaps at no period of our national history has the pursuit of athletics been more generally popular than now. Nor is the practice confined to any particular class; the peasant boy who plays cricket or football on the village green is as much in earnest as the nobleman who plays polo or rides to hounds. At the public schools each boy is encouraged, and, indeed, expected to take part in games; while at the older universities the average undergraduate treats the true athlete with as much respect as he does the scholar. It is not surprising, therefore, that among the thousands who are thus daily employed there are accidental injuries. They present an infinite variety, and for purposes of convenience may be here grouped under two great divisions.

1. One division includes injuries which for the most part give rise to functional derangements of internal organs, temporary in their nature and fitly relegated to the care of the physician. As examples may be mentioned such disabilities as irritable heart, dilated or hypertrophied heart, intermittent and transient hæmaturia, albuminuria, insomnia, and the like.

2. The other, with which I wish to deal to-day, includes gross lesions which are, indeed, the province of the surgeon and result usually from some severe external violence, whereby locomotion or other everyday forms of movement are made difficult or impossible.

The group is a large one, and while some forms of injury are of everyday occurrence, others are so extremely rare that they may be classed among the curiosities of surgery. I will try to interest you during the time at my disposal with some of the injuries most frequently met with. It may be stated in a general way that certain circumstances lead up to these particular disablements, and foremost among these is *the want of proper and careful training*. This want of gradual training applies also to the cases in the other group, that with which the physician is concerned. As an illustration of this I would point out that at the Universities the greatest number of strains and sprains occur during the first fortnight of the October term; the explanation is that during the long vacation the schoolboy, as well as the undergraduate, engages in pastimes which, while useful for the growth and development of the individual, are unsuitable or inadequate as a preparation for the sudden and great strains laid upon muscles and ligaments in such violent forms of exercise as first-class football, polo, and hockey.

Another illustration of the influence of lack of training is the following: I have frequently noticed that boys coming up to the University who have been educated at schools in large towns and accustomed to run in tennis shoes with very flat heels are particularly prone to rupture the tendons and muscles at the back of the thigh as soon as they begin to run on a cinder track, wearing proper running shoes made with spikes and without heels. The reason is obvious: the man who runs in sand shoes does so more with the muscles of his leg than those of his thigh; he runs with flat feet, but as soon as he changes his shoes he sprints on his toes and a different group of muscles, notably the hamstrings, are brought into activity. A similar accident affects those who begin broad jumping with insufficient training. Most of us are familiar with the strain felt in the arm by the effort of throwing a cricket ball early in the season. Beginners at rowing are peculiarly liable to injure their abdominal muscles. Perhaps no muscles in the body require more gradual preparation for sustained effort than do the heart and diaphragm, in spite of their ceaseless activities.

Again, the kind of game is of importance; for while football under the Rugby code accounts for the greater number of injuries, the Association game, speaking generally, is responsible for the most serious of them; contusions, twists, wrenches, and the like are more common with the former, injuries to the viscera are more usually associated with the latter.

Temperature has also an influence. It is well known that rupture of muscle fibres is more common in dry and frosty than in damp and warm muggy weather. Be mindful of the care which a sprinter or high jumper will take to have his limbs rubbed or sponged with warm water to ensure their being warmed before he competes; yet boys and young men think nothing of waiting for half an hour or more on the football field in scanty clothing for the arrival of their opponents. The teaching of physiology accentuates the fact that cold retards muscular contraction and renders muscle tissue less elastic. Whether "frosty weather makes bones more friable" or not, I cannot say, though this is certainly a popular idea.

Athletic injuries result from indirect as well as direct violence, and almost every tissue or viscus may suffer at one time or another. By far the commonest lesions are:—

### Contusions.

These vary in degree from the smallest patch of discoloration in the skin to the most extensive collections of blood. Extravasation sometimes follows the extensive fascial planes of the body and extends far and wide; at others it forms definite local collections, limited after the manner of a cyst. Bruises, though for the most part superficial, are not infrequently deep and involve groups of muscles, are subperiosteal, or even occasionally in such internal organs as the spleen and kidneys. Nor are the serous cavities exempt, for hæmatocele and hæmorrhages under the dura mater and into the pleura and peritoneum are not unknown. The diagnosis of a contusion presents little difficulty. The history of the accident, taken with the general appearances and symptoms, will generally lead to a definite conclusion even where the surgeon has to deal with some deeply placed internal organ.

The principles of treatment are to limit, as much as possible and as quickly as possible, the amount and the extent of the extravasation, and then to promote its complete removal. The application of cold or heat finds most favour with the practitioner of to-day; it is advocated in every text-book and taught in almost every school. Personally, I never now use either of these agents, and for two simple reasons: first, they are efficacious to a very limited extent; secondly, they are seldom conveniently at hand, and are cumbrous and difficult to maintain.

Cold, to be of any real service, must be intense, or immediately applied. It has also the great disadvantages of not being particularly liked by patients and of actually doing harm sometimes to enfeebled persons. Even sloughing and gangrene have followed its prolonged use in albuminuric and diabetic subjects. Similarly hot applications will not check bleeding unless they are hot enough to be intolerable to the skin of the average patient. If the temperature of the application is so reduced as to be comforting and agreeable, bleeding and increased extravasation are encouraged.

Elastic pressure is *par excellence* the satisfactory way of dealing with all forms of extravasation. It is easily applied, ready to hand, comfortable, and possesses the double advantage ot not only being capable of limiting or checking the hæmorrhage, but of also materially assisting its rapid absorption. The treatment of an ordinary black eye is an excellent example of its efficacy. If the patient is seen at once and a pad of cotton wool be applied under a tight bandage, the usual swelling may even be prevented. If, however, swelling has already supervened it will disappear in the space of about six hours if the packing and bandage are carefully applied and retained in position. Not infrequently in packing the orbit thus with wool a small patch of the eyelid or conjunctiva will escape pressure; on removing the bandage some hours later, those parts that have been efficiently packed will present a more or less natural appearance, while that which escaped will be swollen and discoloured. If you take a patient with two black eyes and apply to one of them the ordinary methods of treatment by hot or cold fomentations, and to the other elastic pressure by packing the orbit, the comparison will be so much in favour of the compression plan that you will give up the other.

Contusions of all kinds may be treated on the same plan. Take a large extravasation in the thigh; neither heat nor cold will check the hæmorrhage, but if you apply elastic pressure immediately you will not merely limit but prevent extravasation. When a very large extravasation collects, as under the periosteum of the shin, under the deltoid or quadriceps extensor, or in such loose tissue as that of the axilla, in the sheath of the rectus abdominis, or that between the gastrocnemius and soleus, much time may be saved by aspiration. This should be followed by elastic pressure, and subsequently by massage. If there is reason to believe that clotting has already taken place, you will gain weeks by making a free incision, turning out all clots, and then sewing up the wound. Whether drainage is employed or not, elastic pressure should be applied after the operation, which, it is hardly necessary to say, must be undertaken only with the strictest possible adherence to aseptic principles and practice.

Hæmatoma auris, or football ear, though less frequently seen than formerly, owing to the difference in scrum-formation and the wearing of caps, is responsible for occasional disfigurement. The effusion is between the skin and cartilage of the ear, and generally in the concha. In first and slight cases painting with liquor epispasticus and protection from further injury will generally effect a cure in ten days. In recurrent attacks a free incision should be made into the swelling and the clots turned out; with ordinary care this results in complete cure without deformity and much more quickly than by blistering.

The abdominal viscera are sometimes contused by squeezes or blows. The kidney may be so bruised as to give rise to great tenderness and pain in the loins, followed sometimes by feelings of sickness and faintness and by sharp attacks of hæmaturia. I have met with a considerable number of such cases in football players. The treatment is rest in bed, with low, non-stimulating diet and bland drinks. The hæmaturia, though often severe at first, disappears in twenty-four hours, though it may recur at varying intervals for the best part of a week. Pain is often not a prominent symptom, for which reason it may be difficult to make the patient realise the importance of treatment. The amount of hæmaturia does not appear to influence the ultimate prognosis, for while in cases of copious bleeding the symptom sometimes rapidly disappears, in others of small hæmorrhage it keeps recurring for days.

The testis is frequently bruised by riding, or injured by blows and kicks at football and other games, and a hæmatocele is usually slow in being absorbed. The organ should be immediately suspended after such an injury, and the patient compelled to remain recumbent. When the acute stage is over the testis should be strapped and supported until all swelling has disappeared. When a hæmatocele is large and painful the best treatment is free incision and drainage.

Bruises in the region of the shoulder joint require special mention. Perhaps in no region of the body is wasting more rapid in its appearance, and recovery more tardy. Contusions in this situation are by no means uncommon, and are produced by almost every kind of direct violence. As every movement of the arm is painful, the patient is constrained to keep the joint rigidly fixed. The large subdeltoid bursa is often filled with blood, and the pressure on the muscle exerted by the effusion would seem to affect its nutrition. Whatever the exact explanation is, the fact remains that rapid muscle-wasting with stiffness of the shoulder-joint often results.

As it is somewhat difficult to apply elastic pressure in the usual way to this region, I rest the limb absolutely for about twenty-four hours, and then strap the shoulder as I would any other joint. As soon as the swelling begins to recede and the strapping loosens, I employ gentle massage over the strapping. Passive movements are of quite subsidiary importance; they effect little that massage will not, and are often painful. On the other hand, active voluntary movements should be early encouraged.

### Sprains and Strains.

Next in frequency to contusions are sprains, muscular as well as articular. The very mildest forms of such injuries, in which there is no effusion, are called strains; and in a muscle as well as in a joint every degree of strain may present itself, from simple over-stretching of a muscle or fascia to a partial or complete tearing across.

I am told that these latter accidents are as common amongst the working classes as amongst undergraduates. The diagnosis is, as a rule, very simply made by inspection, and the symptoms are pain, loss of power, and stiffness and swelling from bleeding into the tissues. The fasciæ may be split or torn in such a way as to allow the muscle to herniate, or a tendon may be ruptured or dislocated; all these injuries are grouped with the sprains.

The best way to treat a torn muscle of the thigh, for example, is to apply strapping to the limb. Nothing is more efficacious than to use wide strips of perforated plaster in order, as it were, to support the fasciæ of the thigh and at the same time to divide the muscle into small segments. Do not apply the strapping so as to meet completely round the thigh; leave a space between the ends to allow for the play of the muscles. Instead of laying up such a patient in whom there is no displacement or rupture of an important tendon, I should advise you to allow him to walk, or exercise the limb with a carefully graduated weight and pulley from the very first. What I do in these injuries is to treat them with plaster-strapping only, and after two or three days I allow the man to go back to his rowing, football, or running. I do not believe in resting such cases of simple rupture of muscle fibres with the object of obtaining union; it is idle to expect anything of the sort, for the fibres retract and contract far away from each other. What happens when active movements are allowed is that other fasciculi, or neighbouring muscles, develop and take the place of those ruptured.

In very extensive rupture of a muscle I would advocate open operation and suture, though I do not think the results will ever be quite perfect. Lacerated tendons also should be sutured at once, for they are difficult to deal with in any other way.

The muscles which are most frequently damaged are those of the thigh, the hamstrings as well as the quadriceps extensor, and the deltoid. The hamstrings are sometimes torn close to their pelvic attachment by indirect violence, while the extensors are more frequently injured at their lower connections. Direct violence, such as a kick, sometimes causes an injury to the muscle fibres themselves in the middle of the thigh. The pain in a ruptured muscle is usually insignificant, though numbness may be complained of.

### Articular Sprains.

Sprains of the joints are of frequent occurrence from falls or twists. A previous injury of a similar kind is a frequent predisposing cause, especially if play is resumed at too early a date. The precise lesion which occurs when a joint is sprained varies considerably, and it is no easy matter to make out with accuracy what is the exact state of affairs.

The ligaments suffer most. They may be only overstretched in the mildest cases or they may be partially or completely torn across when the injury is severe. In the worst cases fragments of bone may be detached with them, and since it has become my rule to examine all serious or suspicious cases with the aid of *x*-rays, I have been surprised to find how frequently sprains are complicated by fractures. Many such cases would remain undiagnosed by any other means, and it is therefore of the greatest importance that the surgeon should in all doubtful cases have recourse to radiography. The prognosis should always be very guarded in any case of complicated sprain.

You are all familiar with the symptoms of these lesions: they are pain, disability, and swelling. If the latter is immediate it is due, depend upon it, to hæmorrhage; if deferred twelve or twenty-four hours, to synovitis. The presence of blood within the capsule of a joint excites synovitis, and thus further increases the swelling. Perhaps it will be well for me to deal more with the treatment than with the causes and ætiology of sprains; but let me say just one word on diagnosis. Many people often take a great deal of trouble to make certain of the presence of fluid in the knee-joint, and they rely on floating the patella and other signs; the best way to detect the presence of synovitis of the knee is by simple inspection.

The principles of treatment must be, first and foremost, to check the extravasation of blood into the joint, then to promote the absorption of the material that has been poured out, and, lastly, to restore the strength and mobility of the articulation.

First, I should like to mention that I never by any chance apply a splint or any rigid apparatus to an injured joint. Joints are movable structures, and should never be kept fixed for any length of time. This, I am fully aware, is contrary to accepted teaching, but the principle is applicable to all cases, severe as well as simple. Splints invariably prevent that very movement which ought to take place and, at the same time, that voluntary nerve impulse which prevents not only wasting but also stiffness, and is so necessary to prevent atrophy.

It is of very great importance to begin treatment as soon after the injury as possible. At Oxford I am often fortunate in getting the cases early, and I consider that every hour saved before treatment is commenced shortens the convalescence by days.

I use elastic pressure, because it is applicable to all cases, is efficacious, checks extravasation, and is comfortable. It has been stated by some that the use of elastic pressure is painful and will not be tolerated by patients, but this is rarely the case in my experience.

I have never, I can safely say, had to do with an undergraduate or schoolboy who could not play his games again within three weeks of a sprain, cases complicating fracture alone excluded. I get a large pad of Gamgee tissue or cotton-wool not less than one inch in thickness, and with this I envelop the limb for several inches above and below the joint. Over this a domette or ordinary bandage or one of elastic webbing is applied as tightly as possible, and then fixed. If this is carried out early, and before much effusion has taken place into and around the joint, it is possible to prevent effusion or to limit it considerably. When the injury is severe, and the joint fills at once with blood, the elastic pressure will prevent further effusion.

The principle and the application are alike equally simple, and it is especially important to have plenty of wool and to get uniform pressure everywhere. I remove the bandage in about six hours, and as soon as this is done the skin flushes and the patient feels relief. After reapplying the pressure I then remove the bandage about every twenty-four hours. I never attempt to massage the limb until all effusion into the joint has been got rid of, which is often in a day or two, and should be at the most within a week. I then also advise the patient gently to move his limb, and a good way of attaining this is to tell him to go through various movements with the sound one; as he does this he unconsciously slightly moves the injured limb. Not all the massage and passive movement in the world will prevent the atrophy of disused muscles to which the stimulus of voluntary contraction is not applied. General active movements ought to be commenced a day or two after effusion has disappeared, but although I never use a splint I support the joint for some time by a bandage, which should not be an elastic one.

What are the complications of joint injuries? Subluxations, lesions of nerves, fracture-sprains, and so on. Of the first the most important is internal derangement of the knee-joint. In treating this condition in a man who has suffered from an injury at football, I often order him to take up rowing instead, in order to avoid the recurrence of injury and to develop the extensor muscles of the thigh, by which means the joint is so strengthened that the loose cartilage rarely gives trouble. As for dislocations, I treat them after reduction exactly as I would simple sprains. Fractures complicating sprains do better on passive movements at first.

There are certain sequelæ of sprains which I can do no more than mention. Thus stiff joints, painful joints, wobbly joints, and atrophy of muscles are not rare, but they are preventable by proper treatment. There are others which are not preventable, such as osteoarthritis, osteitis deformans, and occasional deformities, such as knock-knee, or valgus, due to epiphyseal injury.

